# Anodal tDCS over the medial prefrontal cortex enhances behavioral adaptation after punishments during reversal learning through increased updating of unchosen choice options

**DOI:** 10.1093/texcom/tgac006

**Published:** 2022-01-27

**Authors:** Martin Panitz, Lorenz Deserno, Elisabeth Kaminski, Arno Villringer, Bernhard Sehm, Florian Schlagenhauf

**Affiliations:** 1 Department of Neurology, Max-Planck-Institute for Human Cognitive and Brain Sciences, 04103 Leipzig, Germany; 2 Department of Psychiatry and Psychotherapy, Campus Charité Mitte, Charité Universitätsmedizin Berlin, 10117 Berlin, Germany; 3 Department of Child and Adolescent Psychiatry, Psychosomatics and Psychotherapy, University of Würzburg, 97080 Würzburg, Germany; 4 Department of Psychiatry and Psychotherapy, Technische Universität Dresden, 01187 Dresden, Germany; 5 Department of Human Movement Neurosciences, Faculty of Sports Science, University of Leipzig, Leipzig 04109, Germany; 6 Clinic for Cognitive Neurology, University Hospital Leipzig, 04103 Leipzig, Germany; 7 MindBrainBody Institute, Berlin School of Mind and Brain, Humboldt-Universität zu Berlin, 10099 Berlin, Germany; 8 Department of Neurology, Martin-Luther-University of Halle-Wittenberg, 06120 Halle (Saale), Germany

**Keywords:** computational modeling, decision-making, mPFC, reinforcement learning, transcranial direct current stimulation

## Abstract

The medial prefrontal cortex (mPFC) is thought to be central for flexible behavioral adaptation. However, the causal relationship between mPFC activity and this behavior is incompletely understood. We investigated whether transcranial direct current stimulation (tDCS) over the mPFC alters flexible behavioral adaptation during reward-based decision-making, targeting Montreal Neurological Institute (MNI) coordinates *X* = −8, *Y* = 62, *Z* = 12, which has previously been associated with impaired behavioral adaptation in alcohol-dependent patients. Healthy human participants (*n* = 61) received either anodal (*n* = 30) or cathodal (*n* = 31) tDCS versus sham tDCS while performing a reversal learning task. To assess the mechanisms of reinforcement learning (RL) underlying our behavioral observations, we applied computational models that varied with respect to the updating of the unchosen choice option. We observed that anodal stimulation over the mPFC induced increased choice switching after punishments compared with sham stimulation, whereas cathodal stimulation showed no effect on participants’ behavior compared with sham stimulation. RL revealed increased updating of the unchosen choice option under anodal as compared with sham stimulation, which accounted well for the increased tendency to switch after punishments. Our findings provide a potential model for tDCS interventions in conditions related to flexible behavioral adaptation, such as addiction.

## Introduction

Flexible behavioral adaptation is a crucial capacity to survive in dynamic environments. To investigate behavioral adaptation, instrumental reversal learning tasks have been deployed in several studies (e.g. O’Doherty et al. 2001; Cools et al. 2002). During reversal learning, participants learn to choose between 2 different stimuli depending on outcome probabilities. Over the course of the task, outcome contingencies change abruptly and unexpectedly, forcing participants to relearn action-outcome contingencies and, thus, to flexibly adapt behavior. One example to probe behavior in reversal learning tasks on a computational level is reinforcement learning (RL) from observed outcomes, such as rewards or punishments, to repeat or adjust choices (Sutton and Barto 1998). However, humans can adapt their behavior flexibly by also taking into account unobserved outcomes. Hence, on a computational level, flexible behavioral adaptation during reversal learning can be enhanced by counterfactual inference about unchosen choice options (inference about the correlation of reward probabilities). This process constitutes an extension of incremental RL (Li and Daw 2011; Reiter et al. 2016).

Previous work has linked behavioral adaptation during reversal learning to a frontostriatal circuitry comprising the orbitofrontal cortex and the ventral striatum, as well as medial prefrontal regions (Cools et al. 2002; Morris et al. 2016; Izquierdo et al. 2017). The medial prefrontal cortex (mPFC) is suggested to play an essential role in performance monitoring during such tasks (Izquierdo et al. 2017). In particular, the ventromedial prefrontal cortex (vmPFC) has been linked to abstract inferences about higher-order structures during reversal learning (Hampton et al. 2006). It was proposed that the vmPFC encodes the value of the currently chosen option, whereas the anterior prefrontal cortex encodes the value of the unchosen choice option (Rushworth et al. 2011). In a previous study by our research group (Reiter et al. 2016), alcohol-dependent patients exhibited a well-known impairment in flexibly adjusting behavior compared with healthy participants. Computational modeling of behavior revealed reduced updating of the unchosen choice options after punishments as a process underlying the disrupted behavioral adaptation. This impairment was also related to reduced coding of neural error signals incorporating values of the unchosen choice option in the mPFC. However, from such clinical studies, insight is inherently limited to what extent change in mPFC activity is directly involved in modifying behavior.

In the current study, we aimed to investigate a potential causal link between mPFC activity and flexible behavioral adaptation in healthy adults. To this end, we applied transcranial direct current stimulation (tDCS) over the mPFC targeting a coordinate reported to reflect interindividual differences in abstract inference about unchosen choice options in a previous study by our research group (Reiter et al. 2016). TDCS is a noninvasive brain stimulation technique that modulates cortical excitability and allows experimentally controlled inferences about brain activity in a specific area and associated behavior (Nitsche et al. 2000; Kuo and Nitsche 2015). Previous studies have shown behavioral effects on decision-making induced by tDCS over prefrontal regions (Bogdanov et al. 2015; Raja Beharelle et al. 2015; Hämmerer et al. 2016; Soutschek et al. 2018). In the present study, while receiving tDCS, participants performed the same reversal learning task as used in our previous study with alcohol-dependent patients (Reiter et al. 2016). In addition to behavioral analyses of the observed behavior, we sought to investigate the underlying mechanisms of flexible decision-making by the means of computational modeling of RL. Considering the heterogenous effects of tDCS on cognitive functions (Jacobson et al. 2012), we compared both anodal and cathodal stimulation with sham stimulation without an a priori hypothesis about the directionality of possible stimulation-induced effects. Based on the findings of Reiter et al. (2016), we hypothesized that anodal respectively cathodal tDCS would lead to altered updating of the unchosen choice option during RL compared with sham tDCS.

## Materials and methods

### Participants

**Fig. 1:**
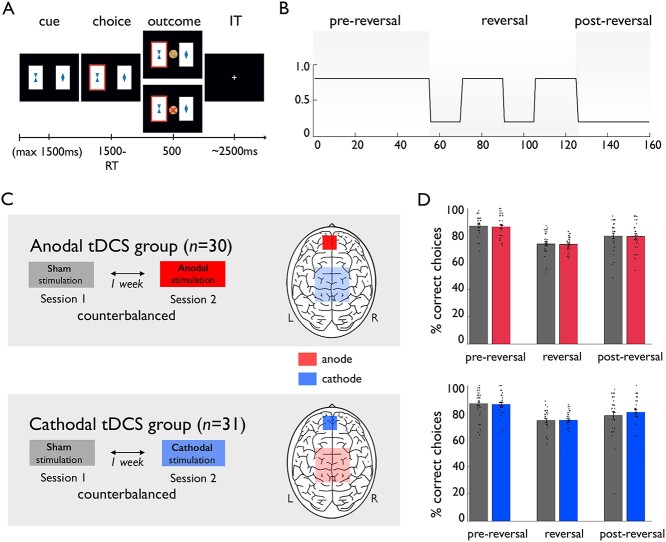
Task and study design; behavioral results on correct choices. A) Reward-based decision-making task. Exemplary trial sequence. B) Anticorrelated structure of the task. One card had a reward probability of 80% along with a 20% probability for a punishment, with inverted reward probabilities for the other stimulus. Reward contingencies were stable during the first 55 trials (“pre-reversal” phase) and the last 35 trials (“post-reversal” phase), whereas during the intermediate 70 trials reward contingencies switched every 15 respective 20 trials (“reversal” phase). C) Study design. Two independent groups (a-tDCS group and c-tDCS group) were recruited and tested in a within-subjects design, undergoing interventions in fully balanced order. D) Behavioral data results on correct choices, i.e. choosing the card with 80% reward probability. In both groups, a significant effect of phase was present, but no significant effect of stimulation was observed. Bar plot figures denote the mean with standard errors of the mean as error bars and individual data points (gray: sham stimulation, red: anodal stimulation, blue: cathodal stimulation).

Sixty-five healthy human participants were recruited. Before participation, volunteers underwent a medical examination to exclude any evidence for neurological diseases or contraindications such as intake of regular medication, including central nervous system-active drugs. Four participants were excluded from analyses due to inadequate task performance (see subsection 2.6.2 Model selection). Consequently, 61 participants were included into analyses, out of which 30 participants were part of an anodal tDCS group, receiving anodal and sham stimulation (15 female, age: *M* = 26.3, SD = 4.1, range = 20–35 years), and 31 participants were part of a cathodal tDCS group, receiving cathodal and sham stimulation (15 female, age: *M* = 27.0, SD = 3.2, range = 22–38 years). Both groups received sham and the respective *verum* stimulation in a fully counterbalanced within-subjects design (Fig. 1C). All participants gave written informed consent before the study and were financially compensated for participation. The study was approved by the local ethics committee of the University of Leipzig.

### Experimental design

Participants were assigned to 1 of 2 groups, which did not differ regarding age, graduation, or verbal intelligence (German vocabulary test; Schmidt and Metzler 1992) (see Table 1). Both groups were tested using a double-blind, sham-controlled, within-subjects design. Importantly, groups differed with respect to the polarity of tDCS received during *verum* stimulation, i.e. anodal or cathodal stimulation, resulting in an anodal tDCS group (a-tDCS group) and a cathodal tDCS group (c-tDCS group). Intervals of as possible 1 week between testing sessions (testing interval: *M* = 7.4, SD = 2.0, range = 6–21 days) avoided potential carryover effects of stimulation. Experiments were performed effectively during the same time in the same subjects between 8:30 AM and 1:00 PM to avoid potential interactions of daytimes. Gender was counterbalanced within groups as was intervention order (sham vs. *verum* stimulation).

**Table 1:** Sample characteristics.

	a-tDCS group (*n* = 30)	c-tDCS group (*n* = 31)	Test statistic
Age	26.3 ± 4.1	27.0 ± 3.2	*t*(59) = −0.71, *P* = 0.48
Gender	15 females/15 males	15 females/16 males	χ^2^(1) = 0.02, *P* = 0.90
Smoking (smoking/non-smoking)	8/22	3/28	χ^2^(1) = 2.98, *P* = 0.084
Graduation (0 = none, 1 = secondary modern school-leaving certificate, 2 = intermediate school-leaving certificate, 3 = university entrance qualification)	3.0 ± 0	3.0 ± 0	
German vocabulary test (verbal intelligence)	34.1 ± 2.5	33.6 ± 2.4	*t*(59) = 0.88, *P* = 0.38
NEO-FFI neuroticism	14.0 ± 4.1	12.7 ± 3.8	*t*(59) = 1.31, *P* = 0.20
NEO-FFI extraversion	20.4 ± 4.1	21.6 ± 3.2	*t*(59) = −1.29, *P* = 0.20
NEO-FFI openness to experience	22.1 ± 3.8	20.5 ± 3.7	*t*(59) = 1.71, *P* = 0.092
NEO-FFI agreeableness	20.5 ± 3.8	19.2 ± 3.8	*t*(59) = 1.30, *P* = 0.20
NEO-FFI conscientiousness	23.4 ± 3.3	24.6 ± 3.6	*t*(59) = −1.36, *P* = 0.18
Barratt Impulsiveness Scale	61.9 ± 8.0	59.3 ± 8.7	*t*(59) = 1.19, *P* = 0.24
UPPS premeditation	32.6 ± 4.4	32.1 ± 3.5	*t*(59) = 0.49, *P* = 0.62
UPPS urgency	34.6 ± 4.7	35.7 ± 4.5	*t*(59) = −0.97, *P* = 0.33
UPPS sensation seeking	26.9 ± 7.0	27.1 ± 8.1	*t*(59) = −0.10, *P* = 0.92
UPPS perseverance	30.6 ± 4.8	32.6 ± 3.8	*t*(59) = −1.82, *P* = 0.074
UPPS total	124.7 ± 13.2	127.5 ± 11.1	*t*(59) = −0.92, *P* = 0.36
BDI-II	6.1 ± 6.1	4.9 ± 4.5	*t*(59) = 0.87, *P* = 0.39
STAI	37.6 ± 8.5	35.8 ± 6.9	*t*(59) = 0.90, *P* = 0.37

Group means are reported with standard deviations; for group comparisons, independent samples *t*-tests or χ^2^ tests were used.

### Decision-making task

While receiving tDCS, participants performed a reward-based decision-making task (Fig. 1A and B) as used previously (Reiter et al. 2016, 2017). Two versions of the task with different stimuli were available and counterbalanced within groups. In 160 trials, participants had to choose between 1 of 2 cards, each represented by a different geometric symbol. Stimuli were randomly assigned to the left or right side. Within the stimulus presentation time (1.5 s), participants had to press a left or right button, after which the selected card was highlighted along with monetary win (10 Eurocent coin) or monetary loss (crossed 10 Eurocent coin) for 0.5 s. A fixation cross was presented during the exponential distributed, jittered intertrial interval (min. 1.0 s, max. 12.5 s). If no response was given on time, the message “too slow” appeared. One of the 2 cards was associated with a high reward probability (80%) and a low punishment probability (20%), whereas the inverted reward probabilities pertained to the other card. Thus, outcome probabilities were perfectly anticorrelated, resulting in a simple higher-order structure of the task. For the first 55 trials (“pre-reversal” phase), the reward contingencies remained stable, whereas during the following 70 trials (“reversal” phase) the reward contingencies switched 4 times, alternating after 15 or 20 trials. During the last 35 trials (“post-reversal” phase), the reward contingencies were stable again. Due to the probabilistic nature of the task, feedback could be either informative or misleading (e.g. getting an informative reward vs. getting a misleading punishment when choosing the option with the 80% reward probability). Because feedback was drawn probabilistically, differences in proportion of the number of informative and misleading events between a participant’s 2 sessions were matched between individuals in both the a-tDCS group and c-tDCS group. On every testing day, participants got standardized instructions about the task with a subsequent training of 20 practice trials without any reversals using a different set of stimuli. Before practicing, participants were informed that one of the two cards had a superior chance of winning money and that they should try to win as much money as possible, as the earned money would be paid out at the end of testing. After the practice trials, instructions ended with a notification that the “better card” could change during the experiment and participants were told to be attentive to such changes. No other information on reversals or the anticorrelated task structure was provided. The reversal learning task used in this study can be broken down to an one-armed bandit task with stable and volatile phases, comparable to studies by Iglesias et al. (2013) and de Berker et al. (2016). Note that in the present task, a reward was received in a win trial and a punishment was received in a lose trial. By contrast, in the task applied in the study by de Berker et al., no punishment was received in a win trial and a punishment was received in a lose trial. Further, in the study by Iglesias et al., in 2 subsamples, a win trial was represented by a reward and a lose trial was represented by omitting the reward, whereas in a third subsample no rewards or punishments were received.

### Transcranial direct current stimulation

**Fig. 2:**
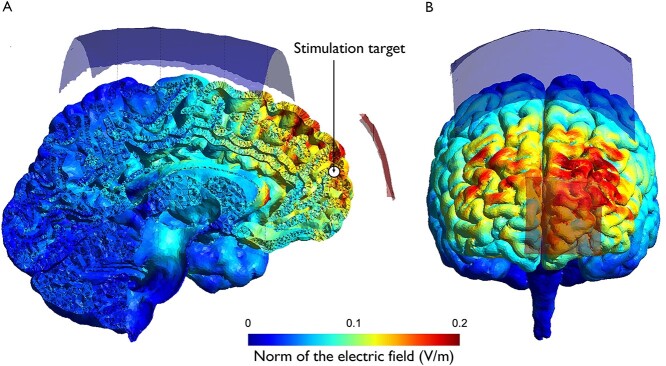
Illustration of electrode positioning and simulation of the induced electric field for anodal stimulation. Simulations show the calculations of the electric field induced by the *verum* stimulation condition in the anodal tDCS group (direct current: 1 mA). A) Lateral view on the left hemisphere congruent with a sagittal plane through MNI dimension *X* = −8. The stimulation target (MNI coordinates: *X* = −8, *Y* = 62, *Z* = 12) is depicted as a white circle outlined in black, marked by a black arrow. B) Frontal view.

For noninvasive brain stimulation, a direct current of 1 mA was delivered for 20 min using a battery-driven DC stimulator (neuroConn, Germany). Target of our stimulation protocol was the mPFC based on its potential key role in enabling flexible value-based decision-making (Fellows and Farah 2003, 2005, 2007; O’Doherty et al. 2003; Rushworth et al. 2009, 2011, 2012; O’Doherty 2011; Glascher et al. 2012). Using neuronavigation (Brainsight Version 2; Rogue Research, Canada) and a standard brain, one stimulation electrode (4 × 4 cm, current density: 0.0625 mA/cm^2^) was placed over the mPFC region targeting MNI coordinates: *X* = −8, *Y* = 62, *Z* = 12, reflecting peak statistics of interindividual differences in coding of neural signatures incorporating values of the unchosen option, which were linked to interindividual differences in behavior, reported in a previous study by our research group (Reiter et al. 2016). The reference electrode (10 × 10 cm, current density: 0.01 mA/cm^2^) was centrally placed on Cz according to the electroencephalography (EEG) 10-20 system. The small size of the stimulation electrode intentionally maximized the current density in the cortical target region in order to promote the desired modulation of cortical excitability, whereas the large size of the reference electrode minimizes current density in the underlying cortex in order to enhance focality of tDCS (Nitsche and Doemkes 2007). The electrodes were attached to the participants’ heads using elastic straps in a pair of saline-soaked sponges. To minimize impedance, the scalp was carefully cleaned with 70% isopropyl alcohol pads before attaching the electrodes to the head. The impedance of stimulation electrodes was always kept below 10 kΩ for each participant. To minimize side effects, such as itching or tingling sensations, current was ramped up and down for 30 s before and after stimulation. During *verum* stimulation, current was delivered for 20 min, while during sham stimulation current was maintained for only 30 s. Five minutes after the onset of stimulation, the experimental task was started. At the end of each testing session, all participants were asked about their estimate whether sham or *verum* stimulation was administered. Successful blinding was tested by questionnaires applying binomial tests on false/right ratings at a *P*-level of 0.05. Figure 2 illustrates the positioning of the electrodes and a simulation of the induced electric field for anodal stimulation. Simulations were based on computations performed by SimNIBS software, version 3.2.1 (Opitz et al. 2015; Thielscher et al. 2015). Using a standard head model, the following parameters were set for simulation: electrode shape = rectangular; electrode size = 4 × 4 cm respectively 10 × 10 cm; electrode type = electrode + sponge; electrode thickness = 2 mm; sponge thickness = 4 mm; sponge size = 4.5 × 4.5 cm. The outcome of the simulation showed that a large area of the prefrontal cortex was affected by tDCS. Though estimated electric field peak values lay mainly in dorsolateral prefrontal areas, simulation illustrated relatively high electric field values in the target region of the mPFC.

### Behavioral data analysis

The behavioral raw data were analyzed within MATLAB (The MathWorks, version 9.1.0.441655, R2016b) with behavioral performance quantified as choices of the stimulus with the 80% reward probability (correct choices). Furthermore, we investigated the effect of previous feedback (reward vs. punishment) on subsequent choices. To this end, we calculated stay behavior depending on the feedback in the previous trial (win-stay vs. lose-stay). Subsequently, the outcome variables correct choices and stay behavior were each analyzed in JASP (JASP Team, JASP Version 0.12.1, 2020) using a mixed-design analysis of variance (ANOVA) with the within-subjects factors stimulation (sham stimulation, *verum* stimulation), phase (pre-reversal phase, reversal phase, post-reversal phase), and, in the case of stay behavior, feedback (reward, punishment) and the between-subjects factor group (a-tDCS group, c-tDCS group). In case of violated assumptions of sphericity, we report Greenhouse–Geisser corrected *F*-values.

### Computational modeling of behavior

To investigate the underlying mechanisms of flexible reward-based decision-making, we formalized learning and decision-making processes via computational models of RL. In RL, values are learned from past experiences and choices are made based on maximized expected value (Sutton and Barto 1998). For all models, expectations are updated via a reward prediction error (RPE) δ:

(1) \begin{equation*} {\delta}_{{\mathrm{Q}}_{\,\mathrm{C}}}^{\left(\mathrm{k}\right)}={\mathrm{R}}^{\left(\mathrm{k}\right)}-{\mathrm{Q}}_{\,\mathrm{c}}^{\left(\mathrm{k}\right)} \end{equation*}

with ${\mathrm{Q}}_{\,\mathrm{c}}^{(\mathrm{k})}$ denoting the expectation for receiving a reward or punishment on trial $\mathrm{k}$ and ${\mathrm{R}}^{(\mathrm{k})}$ representing the actually received outcome. The RPE is used to update expectation of the next trial and is weighted by the learning rate $\alpha$:

(2) \begin{equation*} {\mathrm{Q}}_{\,\mathrm{c}}^{\left(\mathrm{k}+1\right)}={\mathrm{Q}}_{\,\mathrm{c}}^{\left(\mathrm{k}\right)}+\alpha{\delta}_{{\mathrm{Q}}_{\,\mathrm{C}}}^{\left(\mathrm{k}\right)} \end{equation*}

So far, the agent updates expectations for the chosen stimulus only, which we refer to as single update (“SU”). An agent could also update expectations about the unchosen stimulus, thereby learning about the anticorrelated structure of the task. In such a model, which we refer to as double update (“DU”), an increase of the expectation for the chosen card connotes a decrease of the expectation for the unchosen card $u\mathrm{c}$. The expectation for the unchosen stimulus is computed as follows:

(3) \begin{equation*} {\mathrm{Q}}_{\,\mathrm{uc}}^{\left(\mathrm{k}\right)}=1-{\mathrm{Q}}_{\,\mathrm{c}}^{\left(\mathrm{k}\right)} \end{equation*}

The prediction error of the unchosen stimulus ${\delta}_{{\mathrm{Q}}_{\,\mathrm{uc}}}^{(\mathrm{k})}$ is written as:

(4) \begin{equation*} {\delta}_{{\mathrm{Q}}_{\,\mathrm{uc}}}^{\left(\mathrm{k}\right)}=-{\mathrm{R}}^{\left(\mathrm{k}\right)}-{\mathrm{Q}}_{\,\mathrm{uc}}^{\left(\mathrm{k}\right)} \end{equation*}

Thus, the expectation for the unchosen stimulus ${\mathrm{Q}}_{\,\mathrm{uc}}^{(\mathrm{k}+1)}$is updated following:

(5) \begin{equation*} {\mathrm{Q}}_{\,\mathrm{uc}}^{\left(\mathrm{k}+1\right)}={\mathrm{Q}}_{\,\mathrm{uc}}^{\left(\mathrm{k}\right)}+\alpha{\delta}_{{\mathrm{Q}}_{\,\mathrm{uC}}}^{\left(\mathrm{k}\right)} \end{equation*}

To account for possible interindividual differences regarding the extent of updating the unchosen stimulus, we implemented a model where learning about the unchosen choice option is weighted by an additional parameter κ, which we refer to as individually double update (“iDU”) (Reiter et al. 2016, 2017):

(6) \begin{equation*} {\mathrm{Q}}_{\,\mathrm{uc}}^{\left(\mathrm{k}+1\right)}={\mathrm{Q}}_{\,\mathrm{uc}}^{\left(\mathrm{k}\right)}+\kappa \alpha{\delta}_{{\mathrm{Q}}_{\,\mathrm{uc}}}^{\left(\mathrm{k}\right)} \end{equation*}

RL models could include one learning rate α, independent of the kind of feedback received (“1α”). We additionally implemented models with separate learning rates for reward and punishment trials, α_rew_ and α_pun_ (“2α”) (Reiter et al. 2016). For all models, in order to link learning to actual choices, we transformed learned expectations for each trial, i.e. ${\mathrm{Q}}^{(\mathrm{k})}$, to choice probabilities by deploying a logistic function, the softmax:

(7) \begin{equation*} \mathrm{p}\left(\mathrm{a}\right)=\frac{\exp \left(\beta \mathrm{Q}\left(\mathrm{a}\right)\right)}{\sum \exp \left(\beta \mathrm{Q}\left({\mathrm{a}}^{\prime}\right)\right)} \end{equation*}

The softmax equation includes the parameter β, which reflects inverse decision noise, with a high β leading to choices tightly determined by choice expectations and a low β leading to more stochastic choices. Taken together, the model space was formed out of the factors “updating” (SU, DU, iDU) and “learning rate” (1α, 2α), resulting in a total of 6 models (SU-1α-β, DU-1α-β, iDU-1α-β, SU-2α-β, DU-2α-β, iDU-2α-β).

Alternatively, we analyzed a larger model space comprising additional hierarchical Bayesian learning models with a dynamic learning rate (specifically, the hierarchical Gaussian filter, HGF) (Mathys 2011; Mathys et al. 2014) as well as decision models capturing choice repetition independent of learning as comparably applied in a study by Deserno et al. (2020). Because we had a specific hypothesis about updating of unchosen choice options within the RL framework (Reiter et al. 2016), this additional analysis, which was not part of our set of a priori hypotheses, is reported as part of the Supplementary Material.

#### Model fitting

Maximum-a-posteriori estimates of model parameters were derived utilizing the HGF toolbox version 3 (included in the open source software collection TAPAS, https://translationalneuromodeling.github.io/tapas/). For optimization, a quasi-Newton algorithm was applied. Prior means and variances of model parameters were chosen in accordance with previous studies (Reiter et al. 2016; Deserno et al. 2020). See Table 2 for a summary of prior means and variances.

**Table 2:** Prior means and variances of parameters used in computational models.

	Prior mean	Prior variance
Learning models		
α (1α)	0.5	1
α_rew_ (2α)	0.5	1
α_pun_ (2α)	0.5	1
κ (iDU)	0.1	1
Decision model		
β	1	1

#### Model selection

For relative model comparison, we applied random-effects Bayesian model selection (RFX-BMS; [Bibr ref72][Bibr ref72]) using the VBA-toolbox (Daunizeau et al. 2014) to obtain the posterior probabilities (PP), exceedance probabilities (XP), and protected exceedance probabilities (PXP). PP represent the estimated model frequencies of the models considered. The XP represent the posterior probabilities that any given model is more frequent than all others. The PXP adjust XP for the possibility that differences can occur by chance (Rigoux et al. 2014). We examined whether model frequencies were different between the a-tDCS and the c-tDCS group by applying between-groups RFX-BMS, and whether model frequencies were equal across stimulation conditions across both groups by performing within-subjects RFX-BMS. In case of model stability both between-groups and within-subjects, RFX-BMS on the pooled log evidences was applied.

In addition, participants not fit better than chance by any model were identified using a binomial test on the percentage of choices explained by the model derived from negative log-likelihood relative to the number of trials (see Huys et al. 2012; Schlagenhauf et al. 2014) and excluded from the analysis (a-tDCS group: 1 participant, c-tDCS group: 3 participants). Excluding those subjects did not change the results (see Supplementary Material).

To further demonstrate the validity of the model to capture the empirical data, we ran 10,000 simulations of the task per tested session based on the inferred parameters of the winning model and subsequently—using the mean of the respective 10,000 simulations—performed the same analysis on the simulated choice data as on the observed data.

#### Model parameters

Because of our a priori hypothesis that tDCS alters learning about unchosen choice options, we analyzed the learning rate for updating the unchosen choice option α_uc_ resp. α_rew_uc_ and α_pun_uc_ (where, in the case of models with one learning rate, α_uc_ is the product of κ times α and, in the case of models with 2 separate learning rates, α_rew_uc_ and α_pun_uc_ are the products of κ times α_rew_ and α_pun_, respectively) using a mixed-design ANOVA with within-subjects factor stimulation (sham stimulation, *verum* stimulation) and between-subjects factor group (a-tDCS group, c-tDCS group).

### Neuropsychological testing

To assess the influence of tDCS on general cognitive capacities, participants completed a working memory task, the Digit Span Backwards Test (Wechsler 1955), and a task on cognitive speed, the Digit Symbol Substitution Test (Wechsler 1955). The 2 tasks were performed after completing the reversal learning task on both testing sessions. Using JASP, test scores were analyzed by a mixed-design ANOVA with within-subjects factor stimulation (sham stimulation, *verum* stimulation) and between-subjects factor group (a-tDCS group, c-tDCS group), respectively. To ensure that participants in the 2 groups did not differ in important traits and characteristics, we assessed self-report questionnaires on personality traits (NEO-FFI; Borkenau and Ostendorf 2007), impulsivity (Barratt Impulsiveness Scale; Preuss et al. 2008; UPPS Impulsive Behavior Scale; Schmidt et al. 2008), depressive symptoms (Beck Depression Inventory BDI-II; Beck et al. 1996), and anxiety (State Trait Anxiety Inventory STAI; Laux et al. 1981) and applied independent samples *t*-tests for group comparisons (see Table 1).

## Results

### Stimulation protocol

All participants tolerated the stimulation well and blinding was effective as participants could not detect whether they received sham or *verum* stimulation (a-tDCS group: sham stimulation proportion (false/right) = 0.50/.50, *P* = 1.0, anodal stimulation proportion (false/ right) = 0.47/.53, *P* = 0.86; c-tDCS group: sham stimulation proportion (false/right) = 0.52/.48, *P* = 1.0, cathodal stimulation proportion (false/right) = 0.58/.42, *P* = 0.47).

### Behavior in the decision-making task

No significant stimulation effects were observed on choosing the stimulus with the higher reward probability, i.e. correct choices (main effect of stimulation, *F*(1,59) = 0.03, *P* = 0.86; stimulation × group interaction, *F*(1,59) = 0.39, *P* = 0.53; stimulation × phase × group interaction, *F*(2,118) = 0.20, *P* = 0.82). As expected, a significant effect of phase indicated performance differences between the 3 phases of the task (main effect of phase, *F*(1.62,95.73) = 48.59, *P* < 0.001, partial η^2^ = 0.45; see Fig. 1D).

**Fig. 3:**
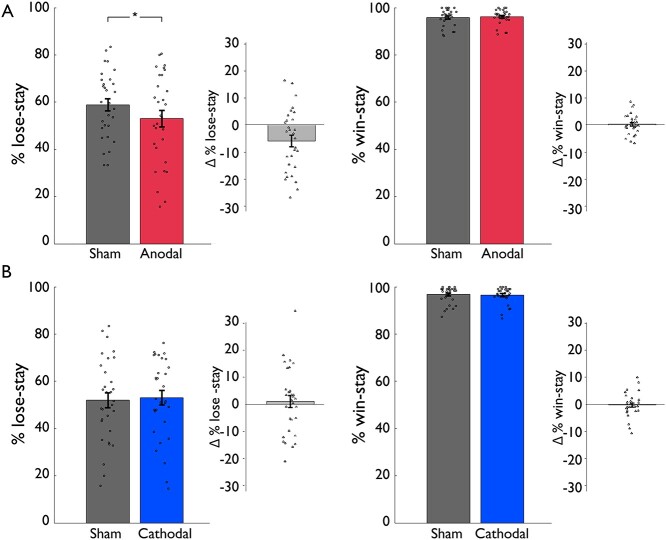
Behavioral data results for feedback-specific stay behavior. In the a-tDCS group A), participants stayed significantly less after punishments during anodal stimulation compared with sham stimulation, while no significant effects were observed in the c-tDCS group B). Displayed are percentages of staying with the same card after receiving a punishment or reward for sham and stimulation condition in both groups as well as the individual difference scores between stimulation (anodal resp. cathodal) and sham condition. Bar plot figures denote the mean with standard errors of the mean as error bars and individual data points; asterisk indicates a significant difference at *P* < 0.05.

Regarding stay behavior, we found a significant stimulation × feedback × group interaction (*F*(1,59) = 5.23, *P* = 0.026, partial η^2^ = 0.08). As expected, a main effect on feedback indicated that participants stayed more with the previous choice after rewards compared with punishments (main effect of feedback, *F*(1,59) = 388.04, *P* < 0.001, partial η^2^ = 0.87). Furthermore, all other main effects or interactions were not significant (stimulation × group interaction, *F*(1,59) = 3.29, *P* = 0.075; all other *P*-values >0.16). To follow up the 3-way interaction, we conducted a repeated measures ANOVA on stay behavior for each group. A significant stimulation × feedback interaction was observed in the a-tDCS group (*F*(1,29) = 5.40, *P* = 0.027, partial η^2^ = 0.16; for all *P*-values see Table 3), whereas no significant effects linked to stimulation were found in the c-tDCS group (all *P*-values >0.30). In the a-tDCS group, post hoc paired-samples *t*-tests revealed that participants stayed less with the previous choice after punishments under anodal stimulation compared with sham stimulation (lose-stay: *t*(29) = 2.74, *P* = 0.010, Cohen’s *d* = 0.50; sham stimulation: mean = 0.59, SD = 0.14, anodal stimulation: mean = 0.53, SD = 0.19; see Figure 3), whereas there was no difference in stay behavior after rewards (win-stay: *t*(29) = −0.45, *P* = 0.66). For an illustration of feedback-specific stay behavior split into the 3 phases of the task, see Supplementary Fig. 1.

**Table 3:** Behavioral data analysis.

Stimulation	*F*(1,29) = 3.64, *P* = 0.066, partial η^2^ = 0.11
Feedback	*F*(1,29) = 185.02, *P* < 0.001, partial η^2^ = 0.86
Phase	*F*(2,58) = 0.29, *P* = 0.75, partial η^2^ = 0.01
Stimulation × feedback	*F*(1,29) = 5.40, *P* = 0.027, partial η^2^ = 0.16
Stimulation × phase	*F*(1.61,46.65) = 2.22, *P* = 0.13, partial η^2^ = 0.07
Feedback × phase	*F*(2,58) = 0.34, *P* = 0.72, partial η^2^ = 0.01
Stimulation × feedback × phase	*F*(2,58) = 2.90, *P* = 0.063, partial η^2^ = 0.09

Repeated measures ANOVA on stay behavior in the a-tDCS group with factors stimulation (sham stimulation, *verum* stimulation), feedback (reward, punishment), and phase (pre-reversal, reversal, post-reversal).

### Computational modeling

**Fig. 4:**
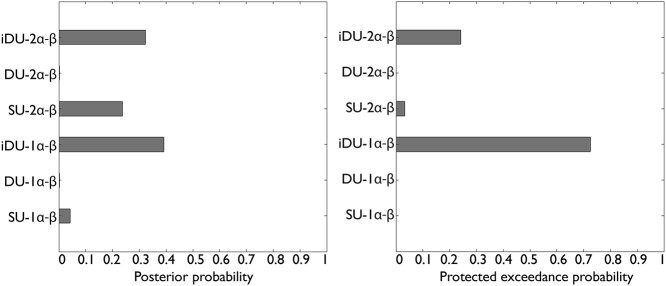
Bayesian model selection. RFX-BMS revealed that a model with individual double updating and one learning rate was the relatively best-fitting model (iDU-1α-β). We show the posterior probabilities and the protected exceedance probabilities of the 6 models. iDU, individually weighted double update; 1α, one learning rate; 2α, separate learning rates for rewards and punishments; β, decision parameter representing inverse decision noise.

Between-groups RFX-BMS showed a high probability that both groups had the same model frequencies (PP = 0.99). Furthermore, in both groups, between-conditions RFX-BMS revealed strong evidence for model stability across stimulation conditions, i.e. within-subjects model stability (a-tDCS group, XP = 1.00, PXP = 0.98; c-tDCS group, XP = 1.00, PXP = 1.00). RFX-BMS on the pooled log evidences revealed that the model with individual double updating and one learning rate was the relatively best-fitting model (iDU-1α-β: PP = 0.39, XP = 0.72, PXP = 0.72; see Fig. 4).

**Fig. 5:**
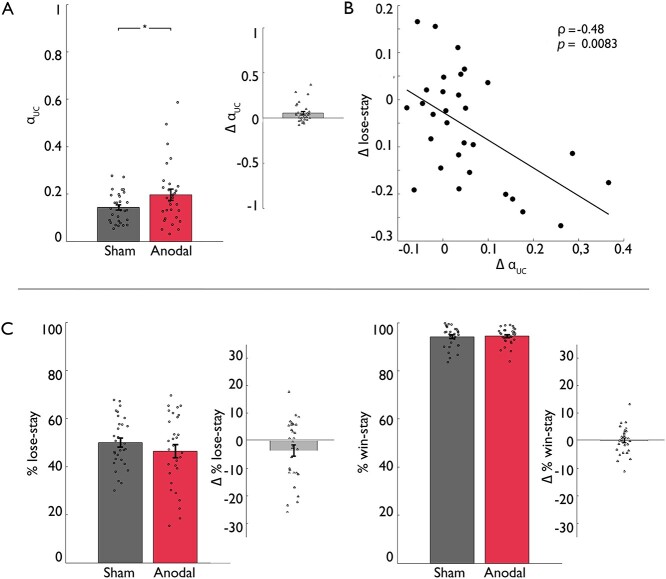
Computational modeling. A) Within-subjects parameter comparison. The parameter α_uc_ was significantly higher under anodal stimulation compared with sham stimulation in the a-tDCS group. Small circles indicate individual values; error bars denote standard errors of the mean; asterisk indicates a significant difference at *P* < 0.05. Δ α_uc_, i.e. the difference score between anodal and sham stimulation regarding α_uc_. Triangles display individual values; error bars denote standard errors of the mean. B) Correlation between computational learning parameter change and observed behavioral change. The change on the parameter α_uc_ correlated with the change on lose-stay behavior induced by anodal stimulation in the a-tDCS group. C) Results for feedback-specific stay behavior on simulated data based on the inferred parameters in the a-tDCS group. The effect of less staying with the previous choice after punishments during anodal stimulation compared with sham stimulation in the empirical data of the a-tDCS group was reproduced with simulated data based on the inferred parameters of the iDU-1α-β model (10,000 simulations per subject and stimulation condition). Bar plot figures denote the mean with standard errors of the mean as error bars and simulated data points.

Regarding the model parameters of the best-fitting model (iDU-1α-β), we observed a significant stimulation × group interaction in the mixed-design ANOVA on the model parameter α_uc_ (*F*(1,59) = 4.93, *P* = 0.030, partial η^2^ = 0.08; main effect of stimulation *F*(1,59) = 4.10, *P* = 0.047, partial η^2^ = 0.07; main effect of group *F*(1,59) = 0.24, *P* = 0.63). Consistent with the behavioral results, follow-up repeated measures ANOVA on the model parameter α_uc_ performed for each group showed a significant effect of stimulation in the a-tDCS group (*F*(1,29) = 7.74, *P* = 0.009, partial η^2^ = 0.21), but no effect of stimulation in the c-tDCS group (*F*(1,30) = 0.02, *P* = 0.88). Post hoc within-subjects parameter comparison using paired-samples *t*-tests or, in the case of non-normality, Wilcoxon signed-rank tests revealed increased updating of the unchosen choice option, represented by model parameter α_uc_, under anodal stimulation compared with sham stimulation in the a-tDCS group (α_uc_: *W* = 350.00, *P* = 0.015, matched rank biserial correlation *r* = 0.51; for completeness, we report within-subjects comparisons of all model parameters; κ: *t*(29*)* = 2.16, *P* = 0.040, Cohen’s *d* = 0.39; all other *P*-values >0.60; see Table 4 and Fig. 5A). In the c-tDCS group, consistent with the absence of a behavioral effect, we observed no significant within-subjects parameter differences (β: *W* = 156.00, *P* = 0.073; all other *P*-values >0.20, see Table 4). Notably, in the a-tDCS group, the change on α_uc_ was significantly correlated with the observed change on lose-stay behavior induced by anodal stimulation (ρ = −0.48, *P* = 0.0083, see Fig. 5B).

**Table 4:** Within-subjects comparisons of model parameters of the best-fitting iDU-1α-β model.

	a-tDCS group			c-tDCS group		
	Sham stimulation	Anodal stimulation	Test statistic	Sham stimulation	Cathodal stimulation	Test statistic
Learning parameters						
α	0.44 ± 0.16	0.47 ± 0.19	*W* = 255.00, *P* = 0.66	0.50 ± 0.17	0.48 ± 0.17	*W* = 186.00, *P* = 0.23
κ	0.33 ± 0.12	0.41 ± 0.15	*t* = 2.16, *P* = 0.040, Cohen’s *d* = 0.39	0.36 ± 0.13	0.37 ± 0.13	*W* = 263.00, *P* = 0.78
α_uc_ (i.e. κ^*^α)	0.14 ± 0.06	0.20 ± 0.13	*W* = 350.00, *P* = 0.015, rank-biserial *r* = 0.51	0.18 ± 0.10	0.18 ± 0.11	*W* = 232.00, *P* = 0.77
Decision parameter						
β	5.98 ± 3.00	5.58 ± 2.33	*W* = 210.00, *P* = 0.66	6.62 ± 3.82	5.60 ± 2.25	*W* = 156.00, *P* = 0.073

Means and standard deviations; paired-samples *t*-tests (*t*-statistics, *P*-values, in case of statistical significance effect sizes by Cohen’s *d*) or, in case of non-normality as examined by Shapiro–Wilk tests, Wilcoxon signed-rank tests (*W*-statistics, *P*-values, in case of statistical significance effect sizes by the matched rank biserial correlation *r*).

Simulated choice data based on the inferred parameters of the best-fitting iDU-1α-β model reproduced the effect on lose-stay behavior in the a-tDCS group (stimulation × feedback interaction, *F*(1,29) = 3.43, *P* = 0.074, partial η^2^ = 0.11; see Fig. 5C).

### Neuropsychology

Analyzing the Digit Span Backwards Test revealed a main effect of group (*F*(1,59) = 5.24, *P* = 0.026, partial η^2^ = 0.08). However, this was qualified by a significant stimulation × group interaction (*F*(1,59) = 4.14, *P* = 0.047, partial η^2^ = 0.07). Repeated measures ANOVA within each group showed a significant main effect of stimulation in the a-tDCS group (*F*(1,29) = 5.72, *P* = 0.024, partial η^2^ = 0.17), whereas we found no significant effect of stimulation in the c-tDCS group (*F*(1,30) = 0.26, *P* = 0.61). In the a-tDCS group, post hoc testing revealed an impairment in working memory as measured with the Digit Span Backwards Test under anodal compared with sham stimulation (*W* = 296.50, *P* = 0.031, matched rank biserial correlation *r* = 0.46, Wilcoxon signed-rank test; sham stimulation: mean = 8.43, SD = 2.64, anodal stimulation: mean = 7.70, SD = 2.20). We found no significant correlation between the change in working memory induced by anodal stimulation and either the change on lose-stay behavior induced by anodal stimulation (*r* = 0.18, *P* = 0.35) or the change on the learning parameter α_uc_ (ρ = −.09, *P* = 0.64) in the a-tDCS group. We observed no significant effects regarding cognitive speed as measured with the Digit Symbol Substitution Test (all *P*-values >0.70).

## Discussion

In the present study, we examined the effect of tDCS over the mPFC on reversal learning in healthy adults. We observed that anodal tDCS modified participants’ adaptive behavior such that participants showed an increased tendency to switch after punishments. Our RL model accounted for this effect by enhanced learning about the respective unchosen choice option under anodal tDCS compared with sham stimulation. In contrast, we observed no behavioral effect for cathodal tDCS compared with sham stimulation. This suggests that flexible behavioral adaptation, specifically lose-stay behavior, and a related specific aspect of learning, namely learning about the unchosen choice option, are directly malleable by anodal but not cathodal tDCS over the mPFC.

In terms of correct choices, we did not find significant differences between stimulation conditions. This may be due to the investigated sample of largely high-educated participants, who performed the task very well. However, in the a-tDCS group, compared with sham tDCS, anodal tDCS induced a subtle but specific change in the way participants managed the task, namely a tendency to switch more frequently after punishments. This finding on lose-stay behavior relates to an important aspect of handling the task that is critical to performance, as dealing with punishments is crucial when differentiating about reversals from stable phases (informative vs. uninformative punishment). Comparison of the computational model parameters suggests an underlying process: increased updating of the unchosen choice option under anodal stimulation compared with sham stimulation. We interpret this as an increase of inference about the alternative choice option under anodal stimulation. After a punishment, a higher parameter κ determines a sharper increased value for the unchosen choice option; thus, the forgone rewarding option becomes more attractive. Put differently, with a higher κ an agent becomes more sensitive to negative feedback through consideration of reward at the alternative choice, which results in an enhanced propensity to switch choices after punishments. At the same time, a higher κ should also lead to a propensity to stay more after rewards. However, in the present study, we found no significant difference regarding “win-stay” in the a-tDCS group, although participants descriptively stayed more after rewards under anodal stimulation. Due to the task structure, win-stay behavior is generally beneficial and a high percentage of win-stay is a prerequisite for a good performance of the task. Furthermore, switching after a win is counterintuitive in such tasks. Participants included in the analysis of the present study indeed followed this strategy, represented in a high, almost at ceiling, percentage of “win-stay,” rendering it unlikely to detect within-subjects stimulation changes. Besides, the present sample was characterized by high education, above average intelligence, and limited age range, which may contribute to this homogenous win-stay behavior. Further studies with participants of different educational levels, intelligence, and age ranges and/or the utilization of a modified task could advance the understanding of potential tDCS effects on win-stay behavior. In addition to RL, we also applied a different modeling approach, namely the HGF framework. While we had a specific hypothesis within the RL framework, the HGF models were performed as add-on analyses, following the application of similar models to the same task in a study by Deserno et al. (2020). In the present study, one of the HGF models showed the best relative model fit, but the specific HGF learning parameters did not account well for the observed behavioral effects (see Supplementary Material). Notably, as described in more detail in the Supplementary Material, the 2 modeling approaches as applied in our study differed with respect to the implementation of updating of the unchosen choice option.

We observed altered lose-stay behavior in the a-tDCS group, which was linked to a value-based learning effect through computational modeling of behavior. This contrasts with a study by Hämmerer et al. (2016), in which anodal tDCS over the vmPFC decreased the percentage of correct choices during value-based decision-making, which was reflected in an increase in the randomness of choices in computational modeling. Different methodological procedures, especially different task design characteristics (e.g. slowly drifting “random walks” reward probabilities), likely contributed to this divergence. On the other hand, the results of the current study are in line with previous work examining the influence of prefrontal tDCS on behavioral adaptation. For example, a study by Raja Beharelle et al. (2015), which used a similar stimulation electrode montage as in our study, showed that tDCS over the right frontopolar cortex (FPC) altered the offset between exploration and exploitation during a reward-based decision-making task. In particular, they found that anodal tDCS lead to higher sensitivity to recent negative prediction errors on exploitative choices, resulting in participants more likely to explore and choose 1 of 2 alternative options. This finding fits well with our behavioral finding that participants were more likely to switch after punishments under anodal stimulation. This similarity is remarkable despite the differences in tasks and modeling. A study by Boorman et al. (2009), which investigated the relationship between adaptive behavior and frontal cortex activity using functional magnetic resonance imaging measures, found that activity of bilateral FPC during a 2-armed bandit task was associated with what they termed “relative unchosen probability.” This term refers to the representation of the relation between unchosen and chosen choice probabilities, hereby gathering inference about the relative advantage in favor of a switch to the alternative choice option. They reported an effect of the “relative unchosen probability” in the FPC to be accompanied by a higher probability to switch to the better choice option. Although the task and modeling were more specifically designed compared with our reversal learning task, the findings resonate well with our modeling approach and finding on learning about the unchosen choice option. As the simulation of the tDCS-induced electric field showed, our tDCS intervention likely modulated cortical activity in the—particularly left—FPC, thereby possibly enhancing constantly made inferences about a potential favorable behavioral switch. In a study by Manuel et al. (2019), tDCS over the vmPFC differentially modulated emotion-induced delay discounting in healthy participants, highlighting the role of the vmPFC in the interplay between reward and emotion. Specifically, they found a tendency towards more impulsive choices following the presentation of positive pictures in high impulsivity trials under cathodal tDCS and reduced impulsivity following neutral pictures in low impulsivity trials compared with both positive and negative induction under anodal tDCS. The study suggests that the investigation of brain–behavior relationship via tDCS interventions may also depend on the emotional context in which the behavior is probed. Future work could use similar paradigms to further investigate the contextual interrelation between emotions and value-based decision-making and the underlying neural mechanisms.

Our finding of altered lose-stay behavior is in line with previous research on behavioral adaptation in regard to serotonergic transmission (Cools et al. 2011). den Ouden et al. (2013) related lose-stay behavior to a genetic polymorphism encoding the serotonin transporter, whereas a polymorphism associated with the dopamine transporter influenced perseveration after reversals. It appears plausible that the current intervention affected the serotonergic circuit, given the role of the mPFC in serotonergic processing, e.g. afferences from the mPFC to the dorsal raphe nucleus (Celada et al. 2001). Previous studies suggest that anodal tDCS enhances functions of serotonergic transmission (Das et al. 2016) and evidence has been found that extracellular serotonin levels affect anodal (and cathodal) tDCS effects on motor cortex excitability (Nitsche et al. 2009). Thus, one might speculate that in the present study, anodal tDCS might have led to altered functioning of the serotonergic system.

Given the often limited options in the treatment of neuropsychiatric disorders, tDCS is a promising, well-tolerated, easy-to-use, and inexpensive tool for therapeutic settings. Indeed, tDCS previously showed beneficial effects in neuropsychiatric conditions such as alcohol dependence (Boggio et al. 2008; da Silva et al. 2013; Klauss et al. 2014), drug addiction (Batista et al. 2015), depression (Mutz et al. 2018), or schizophrenia (Reinhart et al. 2015a, 2015b). Regarding alcohol use disorder, Holla et al. (2020) showed that prefrontal tDCS improved whole-brain network efficiency and increased functional connectivity of a specific prefrontal network in alcohol-dependent patients. However, a recent meta-analysis found no significant effect of tDCS on alcohol-related craving (Mostafavi et al. 2020). Our stimulation target in the mPFC was based on the representations of neural activation differences found in alcohol-dependent patients compared with healthy participants (Reiter et al. 2016). In the study by Reiter et al., on the computational level, alcohol-dependent patients showed an impaired integration of unchosen choice options during reversal learning as reflected in the same parameter as affected by anodal tDCS in the present study. Thus, our results may suggest that anodal tDCS via the mPFC could improve deficits in flexible behavioral adaptation after punishments in alcohol-dependent patients. It should be noted, however, that the observed behavior of the alcohol-dependent patients was considerably impaired compared with the behavior in our present tDCS study. Moreover, the mean values for both age and the estimated learning parameters differed substantially between the 2 studies.

In relation to attention deficit hyperactivity disorder (ADHD), Nejati et al. (2021) showed that anodal tDCS over the right vmPFC coupled with cathodal tDCS over the left dorsolateral prefrontal cortex (dlPFC) enhanced performance during a delay-discounting task and decreased risk-taking behavior in children with ADHD, indicating an interaction between the vmPFC and the dlPFC that is important for reward processing in children with ADHD.

It should be noted that improvements in a particular task achieved via tDCS may not translate into relevant benefits in daily life (e.g. symptoms, recovery) and that intra- and interindividual differences in response to tDCS may be important to consider for successful clinical interventions. Kambeitz et al. (2020) recently presented a promising option to predict tDCS response in patients with an unipolar depressive episode by applying predictive machine learning models based on clinical and neuropsychological characteristics. Effects of tDCS on neurocognitive measures, as in our reversal learning task, could serve as mediators to promote a better understanding of the heterogeneity seen in tDCS treatment effects on symptoms. This could inform why and how tDCS is beneficial in some individuals but not others.

In the current study, we did not observe effects of cathodal stimulation on decision-making behavior. This is in line with other tDCS studies relevant to decision-making, which also found significant behavioral effects for anodal but not for cathodal tDCS (Bogdanov et al. 2015; Soutschek et al. 2018). In general, considering the results of numerous tDCS studies, the former presumption that anodal stimulation always causes excitatory and cathodal stimulation always causes inhibitory effects, is no longer reasonable, particularly regarding cognitive domains (Jacobson et al. 2012; Bestmann et al. 2015; Parkin et al. 2015), supporting the hypothesis that the effects of tDCS on executive functions are multifaceted. For example, in the current study, we observed that anodal stimulation led to an impairment in working memory in the a-tDCS group examined via the Digit Span Backwards Test. Notably, this effect was not related to the behavioral effect seen during reversal learning. Working memory capacity has been associated with anticorrelated activity between mPFC and dlPFC (Keller et al. 2015); thus, anodal tDCS of mPFC might have altered this balance in our study. This further underlines that multiple cognitive processes are likely modulated by tDCS targeting the prefrontal cortex. Further research might apply tDCS designs that combine multiple tasks to examine cross-domain and domain-specific effects of tDCS on executive functions and cognitive processes such as working memory and decision-making.

In the present study, we report a medium effect size (Cohen’s *d* = 0.50) for the observed behavioral difference on lose-stay behavior in the a-tDCS group. Although only a few tDCS studies in the context of decision-making report effect sizes, our effect sizes fit relatively well to comparable tDCS studies albeit some studies report even large effect sizes, e.g. Soutschek et al. (2018) reported a comparable effect size (Cohen’s *d* = 0.46), Manuel et al. (2019) reported medium to large effect sizes (partial η^2^ of 0.11, 0.17, and 0.22), and Ouellet et al. (2015) reported large effect sizes (partial η^2^ of 0.20 and 0.16) for the observed behavioral effects. Notably, Polanía et al. (2018) stated that one might except a high variability regarding effect sizes when placing of the active electrode is based on scalp measurements, which was the case in the latter study by Ouellet and colleagues. In general, the wide variability of tDCS study protocols presents a hurdle in the preparation of meta-analyses (Polanía et al. 2018) and effect sizes reported in current tDCS studies should be interpreted with caution.

Although the target region in the mPFC was carefully selected based on previous research, the stimulation protocol of our experiment has some notable limitations. Firstly, we did not stimulate an active control region in order to better understand the regional specificity of tDCS effects over the mPFC (Polanía et al. 2018). Further, using neuronavigation based on data of individual brain scans instead of one standard reference brain would have enhanced anatomical specificity of the tDCS target region. In addition, an approach to enhance focality of electric fields could be to implement smaller electrodes or high-definition tDCS montages. However, this comes at the cost of increased interindividual variability of the electric fields (Mikkonen et al. 2020). Moreover, in order to better understand the neural signatures underlying the behavioral changes observed in our intervention, research combining stimulation protocols with concurrent neuroimaging methods is warranted. Our study suggests a causal relationship between mPFC activity and specific decision-making processes. However, taking into account the inherent limitations of tDCS, such as restricted focality (Nitsche and Doemkes 2007), and the results of our tDCS simulation, it is likely that the observed effects are not the result of a specific manipulation of mPFC activity, but the outcome of complex physiological interactions caused by neuromodulation of the mPFC and other—in particular dorsolateral—prefrontal areas. Notably, to date, there is a vivid debate in the field regarding the underlying physiological and associated behavioral effects of transcranial electrical stimulation (Liu et al. 2018; Vöröslakos et al. 2018; Khatoun et al. 2019; Krause et al. 2019a, 2019b), e.g. on whether the electric fields induced by common stimulation protocols directly alter the excitability of neurons and to what extent behavioral effects might be due to indirect effects of stimulation such as the involvement of glial cells (Monai et al. 2016) or activation of peripheral nerves (Fertonani et al. 2015).

In sum, the results of our study point towards a causal relationship between choice switching after punishments during reversal learning and prefrontal cortex function manipulated by anodal tDCS over the mPFC. We demonstrate that flexible adaptive behavior and a related specific aspect of learning, namely learning about the unchosen choice option, are directly malleable by anodal tDCS. Our observations could serve as a model for further research aimed at alleviating suffering in patients who exhibit alterations in flexible behavioral adaptation.

## Supplementary Material

Supplement_tDCS_reversal_tgac006
