# Supplementary material for: Anodal tDCS over the medial prefrontal cortex enhances behavioral adaptation after punishments during reversal learning through increased updating of unchosen choice options
Source: Cereb Cortex Commun. 2022 Jan 27;3(1):tgac006. doi: 10.1093/texcom/tgac006 (PMC8874878; doi:10.1093/texcom/tgac006)
Supplement: Supplement_tDCS_reversal_tgac006 [file supplement_tdcs_reversal_tgac006.pdf]

ANODAL TDCS OVER THE MEDIAL PREFRONTAL CORTEX ENHANCES BEHAVIORAL ADAPTATION AFTER PUNISHMENTS DURING REVERSAL LEARNING THROUGH INCREASED UPDATING OF UNCHOSEN CHOICE OPTIONS

Supplementary Material

Martin Panitz[1,2*], Lorenz Deserno[1,3,4*], Elisabeth Kaminski[1,5], Arno Villringer[1,6,7], Bernhard Sehm[1,8], Florian Schlagenhauf[1,2]

[1]Max-Planck-Institute for Human Cognitive and Brain Sciences, 04103 Leipzig, Germany;

[2]Department of Psychiatry and Psychotherapy, Campus Charité Mitte, Charité Universitätsmedizin Berlin, 10117 Berlin, Germany;

[3]Department of Child and Adolescent Psychiatry, Psychosomatics and Psychotherapy, University of Würzburg, 97080 Würzburg, Germany;

[4]Department of Psychiatry and Psychotherapy, Technische Universität Dresden, 01187 Dresden, Germany;

[5]Institute for General Kinesiology and Exercise Science, Faculty of Sport Science, University of Leipzig, Germany;

[6]Clinic for Cognitive Neurology, University Hospital Leipzig, 04103 Leipzig, Germany

[7]Mind Brain Body Institute at Berlin School of Mind and Brain, Humboldt-Universität zu Berlin, 10099 Berlin, Germany

[8]Department of Neurology, Martin-Luther-University of Halle-Wittenberg, 06120 Halle (Saale), Germany

* equal contribution

Corresponding author: Martin Panitz, Department of Neurology, Max-Planck-Institute for Human Cognitive and Brain Sciences, Stephanstrasse 1A, 04103 Leipzig, Germany. Email: panitz@cbs.mpg.de; Phone: +49 341 994000.

Supplementary Methods

Computational modeling of behavior. Alternatively to the model space reported in the main manuscript we analyzed a larger model space including additional hierarchical Bayesian models of learning. In Bayesian accounts of learning, an agent aims to minimize uncertainty, which modulates the weight of experiences and thus the speed of learning. In the present study, we applied the hierarchical Gaussian filter (HGF) (Mathys 2011; Mathys et al. 2014), a generative hierarchical Bayesian model that models multiple, hierarchical organized levels such that a higher-level state influences a lower-level state. In the case of our task, the highest (third) level represents a trial-by-trial computation of the environmental volatility, reflecting the likelihood of a change in action-outcome contingencies at the lower level.

Formally, each state x_i is defined as a Gaussian random walk with the posterior mean expectation $\mu_i^{(k)}$ (cf. $Q_c^{(k)}$ with respect to RL) and variance $\sigma_i^{(k)}$ at each level i, with k being the trial index. The variance $\sigma_i^{(k)}$, i.e., the uncertainty of the estimate at each level, is defined by the next higher level x_{i+1}. The second level follows a Gaussian random walk $x_2^{(k)}$ depending on the previous estimate of $x_2^{(k-1)}$ and the current estimate of the third level $x_3^{(k)}$:

$$(1)\ p\left(x_2^{(k)} \middle| x_2^{(k-1)}, x_3^{(k)}\right) = \mathcal{N}\left(x_2^{(k)}; x_2^{(k-1)}, \exp\left(\kappa x_3^{(k)} + \omega\right)\right)$$

The width of the Gaussian is defined by the parameters κ and ω. While ω captures variance independent of the environmental volatility $x_3^{(k)}$, κ represents the influence of $x_3^{(k)}$ on the second level reflecting the coupling between the levels. As we assume no further level, step size at the third level is defined by the constant parameter ϑ:

$$(2)\ p\left(x_3^{(k)} \middle| x_3^{(k-1)}, \vartheta\right) = \mathcal{N}\left(x_3^{(k)};\ x_3^{(k-1)}, \vartheta\right)$$

As we were primarily interested in higher-level learning and its integration into lower-level learning, we individually estimated ϑ and κ but fixed ω across individuals. The first level denotes participants' predictions for each trial and is defined by a sigmoid transformation of posterior expectations from the second level:

$$(3)\ p(x_1|x_2) = s(x_2)^{x1}(1 - s(x_2))^{1-x1}$$

To derive learning, updates of posterior means at each level i are proportional to the prediction error (PE) from the level below weighted by a precision ratio (cf. equation 4 for the RL equivalent). In short, precision $\pi_i^{(k)}$ is defined as inverse uncertainty (variance) $\pi_i^{(k)} = 1/\sigma_i^{(k)}$:

$$(4)\ \mu_i^{(k)} \propto \frac{\hat{\pi}_{i-1}^{(k)}}{\pi_i^{(k)}} \delta_{i-1}^{(k)}$$

For an exact derivation of precision weights and precision-weighted PE, we refer to the method papers (Mathys 2011; Mathys et al. 2014). In order to test the assumed superiority of three-level hierarchical learning ('3HGF') we additionally applied a reduced two-level variant of the HGF ('2HGF'). Also, we implemented a DU variant of the HGF. By analogy to RL, the expectation about the unchosen stimulus is computed as follows:

$$(5)\ \mu_{i,uc}^{(k)} = 1 - \mu_{i,c}^{(k)}$$

In contrast to RL, we did not include an HGF model that can account for interindividual differences in the degree of updating the unchosen choice option because an individual double-update parameter is not a specific feature of the HGF models and because it seems to be an unfavorable strategy from a computational modeling perspective to include more parameters in the learning model.

In binary choice tasks such as ours, there is strong autocorrelation of choices. Therefore, we implemented the additional decision model parameter ρ, representing choice repetition

irrespective of learned expectations. Learned expectations are transformed to choice probabilities by the softmax as follows:

$$(6)\ p(t,a) = \frac{\exp(\beta\mu_i(t,a)+\rho*rep(r_{t-1}))}{\sum \exp(\beta\mu_i(t,a')+\rho*rep(r_{t-1}))}$$

Here, 'rep' indicates whether the outcome of the previous action was a reward or punishment. Hence, ρ_{rew} and ρ_{pun} represent differences in choice repetition separately for rewards and punishments. In order to avoid overfitting, we either estimated β and fixed ρ_{rew} and ρ_{pun} or we estimated ρ_{rew} and ρ_{pun} and fixed β in our models (we refer to these decision models as 'β' and 'ρ').

Moreover, we aimed to examine the hypothesis that the extent of how tightly choices follow learned expectations might be directly influenced by the trial-by-trial estimates about the environmental volatility. As this requires a measure of volatility enrooted in the observed behavior, we estimated an additional parameter $\mu_3^{(0)}$, representing the initial estimate of volatility at the third level of the HGF, and implemented a softmax in which the trial-by-trial estimates of environmental volatility determine the stochasticity of choices, setting $\beta = \exp(-\mu_3^{(k)})$. As RL and 2HGF do not comprise volatility estimates, this was only applied to the three-level HGF ('3HGF-V').

Taken together, the alternative model space was formed out of the factors 'learning model' (RL-1α, RL-2α, 3HGF, 3HGF-V, 2HGF), 'updating' (SU, DU, iDU), and 'decision model' (β, ρ), resulting in a total of 24 models. See **Supplementary Table 1** for a summary of model parameter priors.

Supplementary Table 1. Prior means and variances of parameters used in computational models of the alternative model space.

	Prior Mean	Prior Variance
Learning models		
RL		
α (1α)	.5	1
α_{rew} (2α)	.5	1
α_{pun} (2α)	.5	1
κ (iDU)	.1	1
HGF		
ω (3HGF + 3HGF-V + 2HGF)	-1.5	0
ϑ (3HGF + 3HGF-V)	-6	1
κ (3HGF + 3HGF-V)	1	1
$\mu_3^{(0)}$ (3HGF-V)	-1	.1
Decision models		
Softmax β		
β	1	1
ρ_{rew}	0	0
ρ_{pun}	0	0
Softmax ρ		
β	1	0
ρ_{rew}	1	1
ρ_{pun}	0	1

Supplementary Results

Behavior in the decision-making task. In order to avoid influences of order of tDCS intervention on behavioral effects, we counterbalanced order of stimulation, i.e., first *verum* then sham stimulation versus first sham then *verum* stimulation. Indeed, no significant influence of order of stimulation on the effect on lose-stay behavior in both the whole sample and the a-tDCS group separately was present (mixed-design ANOVA on stay-behavior with between-subjects factor order of stimulation: stimulation x feedback x group x order of stimulation interaction, $F(1,57) = 2.76$, $p = .10$; repeated-measures ANOVA on stay-behavior in the a-tDCS group with

between-subject factor order of stimulation: stimulation x feedback x order of stimulation interaction, F(1,28) = .43, *p* = .52).

The effect on lose-stay behavior remained significant with all tested participants included in the analysis, including those subjects which were not fit better than chance by any model (mixed-design ANOVA with all *N* = 65 participants on stay-behavior: significant stimulation x feedback x group interaction on stay-behavior, $F(1,63) = 6.27$, $p = .015$, partial $\eta^2 = .09$; repeated-measures ANOVA with all n = 31 participants of the a-tDCS group: significant stimulation x feedback interaction, $F(1,30) = 6.30$, $p = .018$, partial $\eta^2 = .17$, no significant stimulation-linked effects when testing all n = 34 participants in the c-tDCS group, all *p*-values > .25).

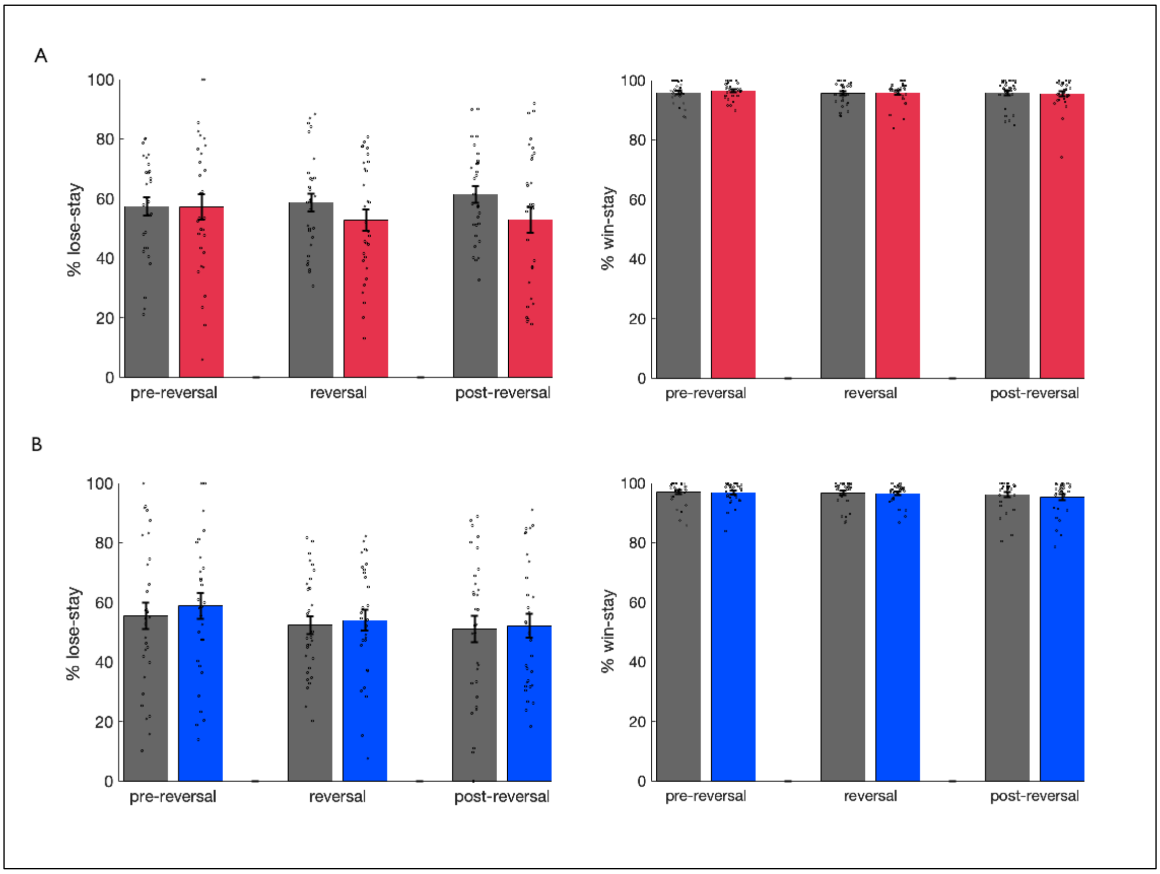

Supplementary Figure 1. Behavioral data results for feedback-specific stay-behavior divided into the three different phases of the task. In the a-tDCS group **(A)**, as shown in the

main manuscript of the paper, a repeated-measures ANOVA on stay-behavior revealed that participants stayed significantly less after punishments during anodal stimulation compared with sham stimulation. Given the statistics of the three-way-interaction stimulation x feedback x phase ($F(2,58) = 2.90$, $p = .063$) and to further illustrate the data, we here additionally show the feedback-specific stay-behavior split up into the three different phases of the task. Explorative post-hoc paired-samples *t*-tests showed that participants stayed significantly less after punishments in the post-reversal phase under anodal stimulation as compared with sham stimulation (lose-stay post-reversal: $t(29) = 2.26$, $p = .03$, Cohen's $d = .41$; lose-stay pre-reversal: $t(29) = .08$, $p = .93$, lose-stay reversal: $t(29) = 2.00$, $p = .054$). In the c-tDCS group **(B)**, no significant effects on feedback-specific stay-behavior dependent on phase were observed. Displayed are percentage of staying with the same card after receiving a punishment or reward for sham and stimulation condition in both groups. Bar plot figures denote the mean with standard errors of the mean as error bars and individual data points.

Computational modeling of the alternative model space. Between-groups RFX-BMS of the alternative model space revealed no group differences (PP that both groups had the same model frequencies ~ 1). In both groups, between-conditions RFX-BMS revealed strong evidence for model stability across stimulation conditions (PXP ~ 1, respectively). RFX-BMS on the pooled log-evidences revealed that a three-level-HGF with double-updating and a decision model capturing choice repetition separately after rewards and punishments and environmental volatility directly linked to decision noise was the best-fitting model overall (3HGF-V-DU-ρ: PP=.43, XP=.8950, PXP=.8950; see **Supplementary Figure 2**). The second best-fitting model was a RL model with individual double-updating and separate learning rates after rewards and punishments (RL-2α-iDU-β: PP=.30, XP=.1050, PXP=.1050; see **Supplementary Figure 2**). Herewith, model selection of the alternative model space confirms previous research, e.g., de Berker et al. (de Berker et al. 2016), that demonstrated the outperformance of HGF models

compared with RL models. Moreover, this is in line with the results of Deserno et al. (Deserno et al. 2020), supporting the assumption that a three-level HGF model linking environmental volatility with choice probabilities accounts well for the observed behavior in our task. Related to the present study, this implies that the outperformance of the HGF compared with RL models was dependent on the specific implementation of the decision model including choice repetition.

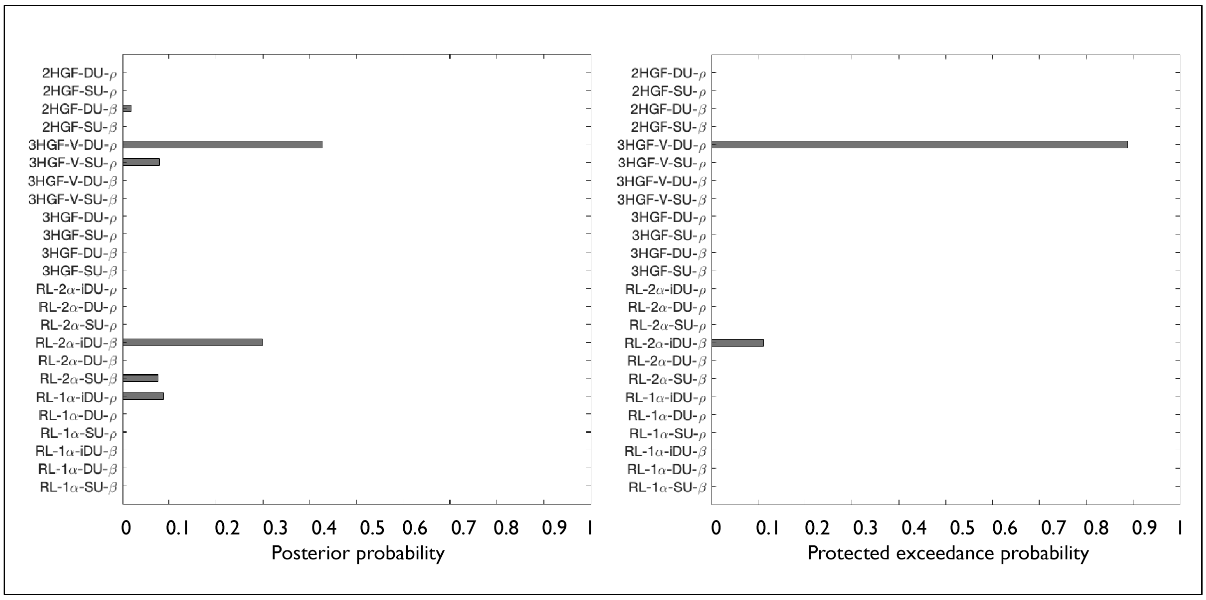

Supplementary Figure 2. Bayesian Model Selection of the alternative model space. Random-effects Bayesian model selection (RFX-BMS) revealed that a three-level-HGF with a decision model capturing choice repetition and environmental volatility linked to decision noise (3HGF-V-DU-ρ) was the relatively best-fitting model. We show the posterior probabilities and the protected exceedance probabilities of the 24 models. Abbreviations: RL-1α = reinforcement learning model with one learning rate; RL-2α = reinforcement learning model with separate learning rates for rewards and punishments; 3HGF = three-level HGF; 3HGF-V = three-level HGF with environmental volatility linked to decision noise; 2HGF = two-level HGF; SU = single-update; DU = double-update; iDU = individually-weighted double-update; β = decision

parameter representing inverse decision noise; ρ = decision model with individually estimated ρ_{rew} and ρ_{pun}, reflecting choice repetition after rewards resp. punishments.

Model parameters. Parameter comparison of the 3HGF-V-DU-ρ model revealed no significant within-subjects differences on learning parameters in the a-tDCS group. In contrast, out of the decision model parameters, a significant difference on the decision parameter ρ_{pun} was present in the a-tDCS group ($t(29) = -3.10$, $p = .004$, Cohen's $d = -.57$, see **Supplementary Table 2**), in line with decreased repetition of choices after punishments under anodal stimulation as compared with sham stimulation. The effect on ρ_{pun} indicates that the winning 3HGF-V-DU-ρ model captured the observed behavior to a substantial extent by learning-independent autocorrelation of choices. Noteworthy, parameter comparison showed a non-significant difference on the learning parameter ϑ ($W = 325.00$, $p = .058$, see **Supplementary Table 2**), reflecting an indication that learning about environmental volatility was enhanced under anodal stimulation as compared with sham stimulation in the a-tDCS group. An increased ϑ suggests that higher-level beliefs about the tendency of the reward contingencies are more sensitive to new information, thus an agent expects to see more change. This could resonate well with the observed behavioral effect to switch more often after losses. However, the near significance finding on ϑ should be treated with caution because learning parameters of the HGF did not cover the behavioral effects significantly and also ϑ showed rather lower recoverability (see Supplementary Material in Deserno et al. 2020).

Parameter comparison of the second best-fitting RL-2α-iDU-β model parameters showed that learning about the unchosen choice option after both rewards and punishments, represented by model parameters α_{rew_uc} and α_{pun_uc} (products of κ times α_{rew} and α_{pun}, respectively), was increased under anodal stimulation in the a-tDCS group (α_{rew_uc}: $W = 346.00$, $p = .019$, matched rank biserial correlation = .49; α_{pun_uc}: $W = 334.00$, $p = .036$, matched rank biserial correlation

= .44; parameter κ: $t(29) = 2.31$, $p = .028$, Cohen's $d = .42$, see **Supplementary Table 2**). Note that the RL-2α-iDU-β model differs from the winning RL model of the original model space reported in the main manuscript only in terms of the learning rate – i.e., separate learning rates for rewards and punishments versus one learning rate. Importantly, parameter comparison of the RL-2α-iDU-β and the winning RL model reported in the main manuscript showed coherent results as both revealed increased updating of the unchosen choice options under anodal stimulation compared with sham stimulation. Consistent with the absence of behavioral effects, we observed no significant parameter differences in the c-tDCS group (see **Supplementary Table 2**).

Supplementary Table 2. Within-subjects model parameter comparison of the best-fitting 3HGF-V-DU-ρ and the second best-fitting RL-2α-iDU-β within the framework of the alternative model space. Means and standard-deviations; paired-samples *t*-tests (*t*-statistics, *p*-values, in case of statistical significance effect size by Cohen's *d*) or, in case of non-normality, Wilcoxon signed-rank tests (*W*-statistics, *p*-values, in case of statistical significance effect size by matched rank biserial correlation).

	a-tDCS group			c-tDCS group		
	Sham stimulation	Anodal stimulation	Test statistic	Sham stimulation	Cathodal stimulation	Test statistic
3HGF-V-DU-ρ						
Learning parameters						
$\mu_3^{(0)}$	-1.05 ± .26	-1.08 ± .24	$W = 196.00, p = .46$	-1.07 ± .27	-1.02 ± .22	$t = 1.01, p = .32$
κ	.63 ± .40	.94 ± 1.01	$W = 280.00, p = .39$	1.13 ± 1.36	1.02 ± 1.09	$W = 240.00, p = .88$
ϑ	-6.006 ± .020	-5.999 ± .020	$W = 325.00, p = .058$	-5.999 ± .017	-6.000 ± .024	$t = -.17, p = .87$
Decision parameters						
ρ_{rew}	.81 ± .49	.76 ± .43	$W = 194.00, p = .44$	.94 ± .41	.90 ± .43	$t = -.41, p = .68$
ρ_{pun}	.07 ± .24	-.05 ± .33	$t = -3.10, p = .004,$	-.09 ± .26	-.06 ± .33	$W = 305.00, p = .27$

			Cohen's d = -.57			
RL-2α-iDU-β						
Learning parameters						
α_{rew}	.51 ± .11	.54 ± .15	t = 1.05, p = .30	.54 ± .13	.54 ± .14	W = 232.00, p = .76
α_{pun}	.42 ± .17	.44 ± .20	W = 250.00, p = .73	.47 ± .19	.46 ± .17	W = 201.00, p = .37
κ	.31 ± .12	.38 ± .16	t = 2.31, p = .028<br>Cohen's d = .42	.34 ± .14	.35 ± .14	W = 255.00, p = .90
α_{rew_uc} (i.e., κ*α_{rew})	.15 ± .07	.20 ±.10	W = 346.00, p = .019, rank-biserial r = .49	.19 ± .09	.19 ± .11	W = 256.00, p = .88
α_{pun_uc} (i.e., κ*α_{pun})	.13 ± .07	.18 ±.15	W = 334.00, p = .036, rank-biserial r = .44	.18 ± .12	.17 ± .13	W = 236.00, p = .82
Decision parameter						
β	6.00 ± 2.97	5.53 ± 2.12	W = 193.00, p = .43	6.48 ± 3.43	5.47 ± 2.07	W = 158.00, p = .08

References

de Berker AO, Rutledge RB, Mathys C, Marshall L, Cross GF, Dolan RJ, Bestmann S. 2016. Computations of uncertainty mediate acute stress responses in humans. Nat Commun. 7:10996.

Deserno L, Boehme R, Mathys C, Katthagen T, Kaminski J, Stephan KE, Heinz A, Schlagenhauf F. 2020. Volatility Estimates Increase Choice Switching and Relate to Prefrontal Activity in Schizophrenia. Biol Psychiatry Cogn Neurosci Neuroimaging. 5:173–183.

Mathys C. 2011. A Bayesian foundation for individual learning under uncertainty. Front Hum Neurosci. 5:1–20.

Mathys CD, Lomakina EI, Daunizeau J, Iglesias S, Brodersen KH, Friston KJ, Stephan KE. 2014. Uncertainty in perception and the Hierarchical Gaussian Filter. Front Hum Neurosci. 8:1–24.
